# A histochemical assay for polyphenolic profiling in cereal grains

**DOI:** 10.18699/vjgb-26-05

**Published:** 2026-03

**Authors:** S.R. Mursalimov, O.Yu. Shoeva

**Affiliations:** Institute of Cytology and Genetics of the Siberian Branch of the Russian Academy of Sciences, Novosibirsk, Russia; Institute of Cytology and Genetics of the Siberian Branch of the Russian Academy of Sciences, Novosibirsk, Russia

**Keywords:** anthocyanin, analytical technique, DMACA, proanthocyanidins, melanins, Fontana–Masson, антоцианы, аналитическая техника, DMACA, проантоцианидины, меланины, реакция Фонтана–Массона

## Abstract

In different cell layers, cereal grains may accumulate various economically important polyphenols such as colored anthocyanins and melanins and colorless proanthocyanidins. To effectively create new cultivars with different combinations of these compounds, a simple, fast, and precise screening method is required. Here, a histochemical assay that includes a combination of hot ethanolic, acidic, alkaline, and ammoniacal silver treatments of grain cryosections followed by microscopy was successfully applied to distinguish these substances in cereal grains. Barley lines previously characterized chemically for the presence of anthocyanins, proanthocyanidins, and melanins in grains were used as a model. In black barley grains, this approach allowed to visually distinguish insoluble melanins that do not react to a pH change from anthocyanins, which can be insoluble or soluble but always react to changing pH. For the first time, ammoniacal silver staining commonly used for melanin identification in human and animal tissues was adapted for melanin identification in plant tissues. Along with melanins, this reagent stains other polyphenols thereby helping to detect colorless polyphenols including proanthocyanidins in the testa of barley grains as confirmed by p-dimethylaminocinnamaldehyde (DMACA) staining. The applicability of this assay to polyphenol profiling was demonstrated not only in the barley grain but also in wheat and common vetch grains. The proposed histochemical assay allows rapid polyphenol screening using a single grain, making it a practical and efficient alternative to time-consuming chromatographic methods for preliminary selection from large sample sets prior to detailed quantitative and qualitative chemical analysis.

## Abstract (ru)

Зерна злаков в некоторых типах клеток могут накапливать разнообразные экономически важные полифенольные соединения, такие как окрашенные антоцианы и меланины, а также бесцветные проантоцианидины. Для эффективного создания новых сортов с различными комбинациями этих соединений в зернах необходим простой, быстрый и точный метод определения их полифенольного профиля. В данной статье для идентификации указанных веществ предложено использовать гистохимический анализ, включающий комбинацию обработок криосрезов зерен горячим этанолом, кислотой, щелочью и аммиачным раствором серебра с последующей микроскопией. В качестве модели использовались линии ячменя, зерна которых ранее были охарактеризованы с помощью аналитических методов на наличие антоцианов, проантоцианидинов и меланинов. В черных зернах ячменя данный подход позволил отличить нерастворимые меланины, не реагирующие на изменение pH, от антоцианов, которые могут быть растворимыми или нерастворимыми, но всегда меняют окраску при изменении pH. Впервые обработка аммиачным серебром, широко применяемая для идентификации меланина в тканях человека и животных, была адаптирована для использования в растительных тканях. Наряду с меланинами, этот реагент окрашивает и другие полифенолы, что позволяет выявлять в том числе бесцветные соединения. С помощью этого подхода были выявлены проантоцианидины в оболочке зерна ячменя, что подтверждено окрашиванием 4-(диметиламино)циннамальдегидом (DMACA). Разработанный протокол успешно применен для определения полифенольного профиля зерен ячменя, пшеницы и вики посевной. Метод позволяет проводить быструю оценку полифенольного профиля с использованием единичных зерен, что является эффективной альтернативой трудоемким хроматографическим методам на этапе предварительного отбора коллекционного материала перед его детальным химическим анализом.

## Introduction

In different cell layers, cereal grains may accumulate different polyphenols such as colored anthocyanins and melanins and colorless proanthocyanidins. Melanins and anthocyanins are known to participate in protection of grain against severe environments and threats by predators and pathogens (Winkel- Shirley, 1998; Glagoleva et al., 2020), while proanthocyanidins are conducive to dormancy and longevity of seeds (Debeaujon et al., 2000).

The aforementioned polyphenols are industrially important; they affect final application of grains as a raw material. For example, due to their protein-binding capacity leading to chill haze (reducing beer quality), proanthocyanidins synthesized in the testa (i. e., the seed coat) of barley under the control of the Ant28 gene are undesirable in malt cultivars (von Wettstein, 2007). Anthocyanins that accumulate either in the aleurone layer or in the pericarp under the control of genes Blx1–5 or Ant1 and Ant2 cause the grain color to be blue and purple, respectively, and melanins that accumulate in husks and the pericarp under the control of Blp1 cause the grain color to be black (Shoeva et al., 2018). These anthocyanins and melanins are promising functional food ingredients and are desirable in cultivars for human consumption (Matseychik et al., 2020; Loskutov, Khlestkina, 2021). Currently, the generation of new cultivars with distinct compositions of polyphenolic pigments in grain is an advanced task for plant breeders. To reach this goal, analysis of many specimens for certain compounds to choose adequate donors carrying the desired genes and screen the resulting hybrids is required.

Currently available methods of chemical profiling of polyphenols such as high-performance liquid chromatography (HPLC) or thin layer chromatography (TLC) allow to separate a large number of individual compounds (Vermerris, Nicholson, 2008), but they are unnecessarily time-consuming for preliminary screening of many specimens. In addition, they do not show histological patterns of polyphenol accumulation, which are known to be unevenly distributed within cereal grain and should be taken into account while processing grain for food (Barron et al., 2017). As an alternative to these methods, microscopy techniques can be used (Panato et al., 2017).

Among polyphenols, anthocyanins can be easily detected by microscopy owing to their color, which may vary from red to blue and their shades and depends on the chemical structure of the molecules, the presence of copigments, and metal ions (Davies, 2009). Change of color at different pH levels is a wellknown characteristic feature of anthocyanins. In a strongly acidic medium (pH < 1), anthocyanins are in the cationic form, having a red color, whereas between pH 4 and 6, the cation loses two protons and turns blue (Davies, 2009). This pH-dependent color change of anthocyanins has been adapted to the detection and quantification of these compounds (Lee et al., 2005; Wrolstad et al., 2005).

Melanins are dark brown to black pigments formed in plants by oxidation of diverse phenolic precursors among which catechol, caffeic, chlorogenic, protocatechuic, p-coumaric and gallic acid have been reported (Bell, Wheeler, 1986; Solano, 2014; Varga et al., 2016). The polymeric nature and poor solubility of melanins constrains research on their chemical structure. In plants, melanins are the least studied group of pigments unlike melanins of the other kingdoms of living organisms, such as animals, bacteria, and fungi melanins, which were surveyed in details at the biochemical and molecular genetic levels (Britton, 1983; Solano, 2014; Glagoleva et al., 2020). It has been shown that melanins in barley grains accumulate in specific plastids called melanoplasts (Shoeva et al., 2020; Mursalimov et al., 2022)

In barley, it can often be challenging to distinguish visually dark melanins from anthocyanins owing to a brownish hue they acquire during grain maturation (Glagoleva et al., 2022b). Furthermore, anthocyanins and melanins can accumulate in one grain simultaneously (Glagoleva et al., 2022a). Melanins are usually identified by a series of solubility tests, and spectroscopy techniques such as Fourier transform infrared (FT– IR), nuclear magnetic resonance (NMR), and the electron paramagnetic resonance (EPR) spectroscopy (Glagoleva et al., 2020). They dissolve in alkali solutions, discolor under the influence of strong oxidizing agents, react with FeCl3 and are stable in common organic solvents that have been reported as hallmarks of melanin (Sava et al., 2001). Histochemical analysis of the melanin pigments in plant tissues is not common. For detection of melanins in human and animal tissues, the Fontana– Masson (FM) protocol with ammoniacal silver staining is commonly used. The protocol is based on the reduction of ammoniacal silver to metallic silver by phenolic substances. The product of the reaction is an insoluble black precipitate, which can be identified on tissue sections by light microscopy (Wildi, 1951; Bancroft, Gamble, 2008).

Here, we use the barley lines, previously characterized chemically for the presence of anthocyanins, proanthocyanins, and melanins, to demonstrate the ability of a new histochemical assay to differentiate these substances in grain tissue. Among the lines used in the study are Bowman (hereafter: Bw) backcross- derived near-isogenic lines (NILs) developed for the presence of anthocyanin (i:BwAnt1Ant2, hereafter: PLP) and melanin (i:BwBlp1, hereafter: BLP) compounds chemically identified by HPLC, a series of solubility tests, and Fourier transform infrared spectroscopy (Shoeva et al., 2020; Glagoleva et al., 2022c). The lines have been created by introgression of recombinant genetic fragments from colored donor lines into the genetic background of the parental cultivar Bowman, which lacks pigmentation in the grain (Druka et al., 2011). Another line used in the study is the mutant line ant25.264, which lacks proanthocyanidins in the grain. This line was induced by chemical mutagenesis of the parental cultivar Secobra 18193, which retained its ability to accumulate proanthocyanidins in the grain (Jende-Strid, 1993). NILs and mutant lines, which have relatively few genetic differences from their parental cultivars, and are used extensively in genetic studies aiming to discover the association of phenotypic differences between the lines with specific chromosome regions or nucleotide polymorphisms. The use of these lines in studies of the genetic control of the synthesis of different classes of polyphenolic compounds in barley grains has been accompanied by their chemical profiling (Jende-Strid, 1993; Shoeva et al., 2020; Glagoleva et al., 2022c

Here, taking advantage of the detailed characterization of these lines, a histochemical assay was developed to identify the different classes of polyphenolic compounds in barley grains. The described protocol may be employed for fast screening of a large number of samples and requires only a single grain for every sample. The method could be adapted to the grains/ seeds/fruits of a wide variety of plant species. Here, as an example, besides barley, we successfully applied the assay to polyphenolic pigment profiling of black grains of wheat line s:S29Ba14Th(4D)Pp3PPp-D1PF (hereafter: S29BLACK) accumulating anthocyanins in the pericarp and aleurone layers simultaneously (Gordeeva et al., 2022), and common vetch cultivar Obskaya 16, having a black color of grain due to accumulation of anthocyaninis in the macrosclereids (Goncharova, 2020; Mursalimov et al., 2021).

## Materials and methods

Plant material and growing conditions. The plant genetic resources utilized in the current study and their origin are listed in Table S11. To obtain seeds for developing the protocol for histochemical polyphenolic profiling, cereal plants were grown in a greenhouse of the ICG SB RAS (Novosibirsk, Russia) under a 16 h photoperiod at a temperature range of 20–25 °C, whereas vetch was grown in an experimental field of the ICG SB RAS (Novosibirsk, Russia) in 2021.

Supplementary Materials are available in the online version of the paper: https://vavilovj-icg.ru/download/pict-2026-30/appx4.pdf


The histochemical assay. The grains were sampled at the hard dough developmental stage (BBCH-87) from each of the aforementioned barley and wheat lines and at the brown pod stage for vetch grains. All of them were snap-frozen in liquid nitrogen and stored at –70 °C until analysis. Prior to sectioning, the frozen grains were kept at −20 °C for 30 min, mounted, and embedded in the Tissue-Tek O.C.T.TM compound medium (Sakura Finetek Europe B.V., Netherlands). The sectioning was carried out at −20 °C using an HM 505N cryostat microtome (Microm, Germany). Sections of 20 μm thickness were mounted on a poly-L-lysine slide (Thermo Fisher Scientific, Germany) and fixed by the addition of 1 mL of a precooled fixative (4 °C) onto the slide. Then, the slides were air dried at room temperature. For sections prepared for hot ethanol treatment, 80 % ethanol in water was utilized as a fixative, and 8 % formaldehyde (Sigma-Aldrich, USA) solution in phosphate-buffered saline (pH 7.4) was used as a fixative for the rest of the samples. All the slides were next rinsed for 15 min in distilled water to remove the fixative and embedding medium. After that, for hot ethanol treatment, the slides were transferred into 80 % (v/v) ethanol in water at 80 °C for 60 min incubation. The slides were then rinsed in distilled water and mounted in glycerol. For the pH test, the slides were immersed for a few seconds either in 1 M NaOH or 1 M HCl, quickly rinsed in distilled water and mounted in glycerol.

For ammoniacal silver staining, the FM protocol (Bancroft, Gamble, 2008; Kiernan, 2008) was performed. A stock solution was prepared by adding ammonium hydroxide solution drop by drop to a 10 % (w/v) silver nitrate water solution, until the solution yielded a precipitate and cleared again. After that, a 0.01 % working solution was prepared by dilution with distilled water. Next, the slides with the sections were incubated in the working solution for 15 min at 60 °C. The slides were then rinsed in distilled water and incubated in a 5 % (w/v) sodium thiosulfate water solution for 15 min, rinsed in distilled water again, and mounted in glycerol. The sections were analyzed under an Axio Observer Z1 microscope equipped with an AxioCam HRc camera (Zeiss, Germany). The ZEN software was used for image analysis and processing (Zeiss, Germany). The microscopic analysis was carried out at the Multi-Access Center for Microscopy of Biological Objects at the Institute of Cytology and Genetics, SB RAS (Novosibirsk, Russia). For each variety, at least three independent grains were analyzed, and a minimum of ten sections were examined for each grain.

Dimethylaminocinnamaldehyde (DMACA) staining. The sections fixed as described above were incubated overnight in a fresh solution of 0.5 % (w/v) DMACA (Sigma-Aldrich, Germany, kindly provided by Dr. Pavel Zykin from St. Petersburg State University, Russia) in 6 N HCl. The microscopic analysis was carried out as described above. For each variety, at least three independent grains were analyzed, and a minimum of ten sections were examined for each grain

## Results

**Fig. 1.**
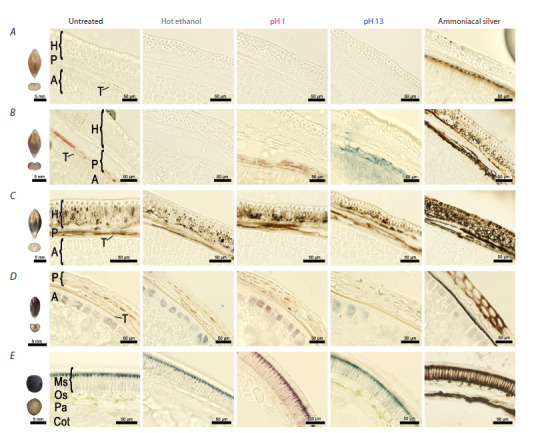
Seeds and cross-sections of the seeds of barley, wheat and vetch after corresponding treatment (brightfield microscopy). Horizontal lines: A – Bowman, B – PLP, C – BLP, D – S29BLACK, E – vetch. A: aleurone layer, H: husk, P: pericarp, T: testa, Cot: cotyledon, Ms: macrosclereids, Os: osteosclereids, Pa: parenchyma

Grain sections of three barley lines were analyzed by the proposed assay: the control unpigmented Bowman line, the PLP line with purple grains, and the BLP line with black grains. Five groups of sections were obtained for every grain: untreated or treated with hot ethanol, HCl, NaOH, or ammoniacal silver. All the resulting specimens were examined by brightfield microscopy (Fig. 1).

Grain tissues on sections of the control Bowman line did not have any visible pigmentation at the hard dough stage (Fig. 1A). There was no visible staining after hot ethanol treatment as well as after the pH change. The only noticeable dark staining appeared after ammoniacal silver treatment of the testa.

Untreated sections of the PLP line are characterized by accumulation of purple pigments in cells of the pericarp (Fig. 1B). The other cell types of this line do not have any pigmentation. The purple color disappeared totally after hot ethanol treatment, and the whole slice became colorless. At pH 1, the purple color persisted, whereas at pH 13, it turned bluish-green and notably leached into surrounding tissues. Intensive dark staining was observed in the pericarp, testa, and husk after ammoniacal silver treatment.

In the BLP line, brownish-black pigments were observed in untreated sections in the pericarp and husk (Fig. 1C). This pigmentation did not change after hot ethanol treatment or after the pH changes. After ammoniacal silver treatment, the brownish-black pigmentation became even more intense in the pericarp and husk, where it was present before, and it emerged in the previously colorless testa and upper epidermal cells of the husk

The sections prepared from wheat and vetch grains were assayed in the same manner. In black grains of wheat line S29BLACK, pale bluish-gray and purple pigments were revealed in aleurone and pericarp cells, respectively (Fig. 1D). The pigmentation did not disappear after hot ethanol treatment, and the bluish-gray color turned blue. At pH 1, blue aleurone cells became purple, while the purple pericarp cells did not change their color. At pH 13, the purple color of pericarp cells became blue, while the bluish-gray color of aleurone cells changed to bluish-green. Ammoniacal silver treatment gave rise to gray pigmentation in the aleurone, a brownish-black color in pericarp cells, and an intensive black color in the testa. It should be pointed out that only in wheat grains was the brownish-black color detected in cell walls of the pericarp.

In black vetch grains, blue pigmentation was observed in macrosclereids (Fig. 1E), becoming more intense after hot ethanol treatment and failing to be washed out. The blue pigments turned purple at pH 1 and became bluish-green at pH 13. In macrosclereids, ammoniacal silver stained the blue pigments brownish-black. In addition, the brownish-black pigmentation emerged in previously colorless parts of macrosclereids as well as in osteosclereids and cells of the parenchyma

**Fig. 2.**
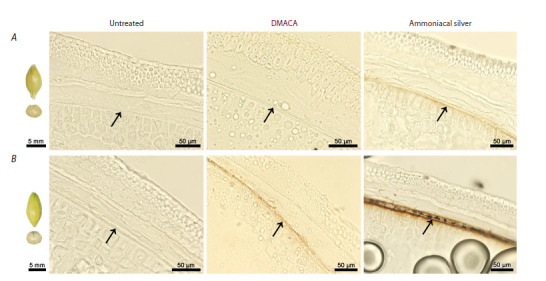
Seeds and cross-sections of the seeds of anthocyanin-free (A) and anthocyanin-rich (B) grains of barley after DMACA and ammoniacal silver staining (brightfield microscopy). Horizontal lines: A – ant25.264, B – Secobra 18193. Arrows indicate testa

A comparison between ammoniacal silver staining and DMACA staining for revealing colorless polyphenols was performed on proanthocyanidin-less barley mutant line ant25.264 and its parental cultivar Secobra 18193 containing proanthocyanidins in grains (Fig. 2).

There was no noticeable staining after the DMACA treatment of grains of ant25.264, whereas pale brown staining appeared in testa after ammoniacal silver treatment (Fig. 2). The brownish-purple staining was observed in the testa of the parental Secobra 18193 grains after DMACA treatment, and in the same tissue, intense black staining was registered after ammoniacal silver treatment (Fig. 2).

**Fig. 3.**
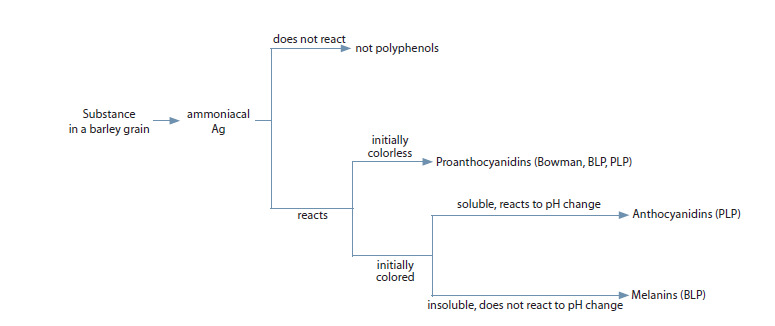
The scheme for identification of polyphenols in barley grains.

After summarizing the results of all the above-mentioned tests, we built a scheme for identifying anthocyanins, melanins, and proanthocyanidins in barley grains (Fig. 3).

## Discussion

The analysis of polyphenolic profiles of crops is quite important for both the elucidation of fundamental aspects of polyphenol biosynthesis and for industrial applications of the raw material. Some attempts have been made to find a fast and simple way to perform polyphenolic profiling of a large number of samples, such as high-throughput phenotyping approaches based on image analysis of color characteristics (ElMasry et al., 2019; Komyshev et al., 2023). Nevertheless, histochemical techniques combined with microscopy seem to be the only approach nowadays allowing a fast and precise polyphenolic profiling on a limited amount of the experimental material and providing information about the cell type where these polyphenols accumulate. Our assay involving a series of standard chemical tests applied to cryosections followed by microscopic examination successfully showed the possibility of identifying polyphenolic pigments in barley, wheat, and vetch grains

Hot ethanol treatment, which is a common test for solubility (Takahashi, Hara, 2014), allowed us to easily distinguish soluble anthocyanins in PLP grains from insoluble melanins in BLP grains. It is known that brown-pigment melanins necessitate a special harsh treatment to become soluble and are insoluble under the hot ethanol conditions we used (Sava et al., 2001). Nevertheless, not all anthocyanins are soluble. Melanins can be distinguished from insoluble anthocyanins by the absence of a color transition at changing pH, which is a well-known characteristic feature of anthocyanins (Davies, 2009).

Thus, in the PLP line, the solubility and pH tests enabled us to identify the purple pigments – that completely disappeared during hot ethanol treatment and changed color to blue in the presence of sodium hydroxide – as anthocyanins, and to identify the BLP line’s black pigments – that did not react to hot ethanol treatment and changes in pH – as melanins. The same results have been previously obtained by HPLC, a series of solubility tests, and Fourier transform infrared spectroscopy, when extracts from PLP and BLP grains have been assayed (Shoeva et al., 2020; Glagoleva et al., 2022c).

As a first step, a FM protocol was included in our histochemical assay to confirm the presence of melanins, which do not react to hot ethanol treatment and a pH change. The FM protocol is commonly used for the detection of melanins in human and animal tissues (Bancroft, Gamble, 2008; Kiernan, 2008) and has not been used previously for the detection of melanins in plant tissues. It turned out that the FM protocol allows the detection of not only melanins by staining them deep black but also of other polyphenols in a grain, including colorless ones, owing to their common ability to reduce ammoniacal silver to metallic one. It is known that silver nitrate reacts with aromatic compounds that contain two or more phenolic hydroxyl groups (Wildi, 1951).

In the barley grain, black melanins, purple anthocyanins, and colorless proanthocyanidins can be detected by ammoniacal silver staining. In grains of the BLP line, the melanincontaining tissues (pericarp and husk) were expectedly stained by ammoniacal silver; however, metallic silver was also noted in the testa, implying the presence of hidden polyphenols. Ammoniacal silver staining in the BLP line was the strongest in comparison to lines Bowman and PLP, thus pointing to the presence of a much greater amount of polyphenols in BLP. The higher accumulation of phenolic compounds in the BLP line has also been observed elsewhere by HPLC analysis (Shoeva et al., 2016). Moreover, for the black-grained BLP line, the highest antioxidant activity has been documented (Glagoleva et al., 2017). The ability of melanins and colorless polyphenols to reduce ammoniacal silver may be explained by their wellknown antioxidant properties (Quideau et al., 2011; Panzella et al., 2012). In the PLP line, ammoniacal silver strongly stained both visible anthocyanin pigments and previously colorless tissues, but less strongly than in the BLP line. In all three barley lines, ammoniacal silver stained colorless polyphenols in the testa. It was especially noticeable in noncolored control Bowman grains, which do not contain any visible pigments and do not react to hot ethanol and to changes in pH but show prominent black granules after ammoniacal silver staining in only one tissue: the testa. It is in this tissue of the barley grain that colorless proanthocyanidins are synthesized (Jende-Strid, 1993).

To evaluate the efficiency of ammoniacal silver staining at detecting proanthocyanidins, it was compared with the DMACA staining. Both methods were applied to barley grain sections enriched with and low in proanthocyanidins. We observed the better sensitivity of ammoniacal silver staining: DMACA did not detect any proanthocyanidins in proanthocyanidin-less ant25.264, while the ammoniacal silver reaction in its turn was weak but positive. It is also important to state that DMACA staining is performed in 6 М HCl, and HCl negatively affects tissue preservation. Moreover, owing to the low pH of the DMACA solution, this reagent must not be used in the presence of anthocyanins because they could change their color under such conditions and yield a false positive result. Due to the aforementioned problem, we could not apply DMACA staining to the detection of proanthocyanidins in grains of PLP barley, S29BLACK wheat, and vetch. The results allowed us to conclude that ammoniacal silver staining has some advantages against DMACA staining and could replace it to locate proanthocyanidins in grains.

The observations made on the barley grain sections treated with hot ethanol, HCl, NaOH, and ammoniacal silver helped us to develop a protocol for the identification of polyphenolic pigments in barley grains; it is presented as a scheme in Figure 3. The relevance of the protocol was confirmed by chromatography and independent chemical tests reported previously for the same barley lines (Shoeva et al., 2020; Glagoleva et al., 2022c). It is worth noting that this protocol was devised for barley. For polyphenolic profiling of grains of other plant species in the same manner, it is desirable to verify the reliability of the proposed histochemical techniques by precise analytical chemistry approaches. Nevertheless, for a preliminary analysis, the proposed assay could be used in its current state even for different species. Here we extended the application of this assay to polyphenol profiling of darkly pigmented grains of wheat and vetch. Wheat line S29BLACK was constructed by marker-assisted selection for the presence of genes ThMyc4E and Pp, which control the synthesis of blue and purple anthocyanins in the aleurone and pericarp (Gordeeva et al., 2022). The black color of grains of the vetch line we analyzed in the current work has been ascribed to the blue pigments accumulating in macrosclereids; however, the precise chemical composition has not been determined (Mursalimov et al., 2021).

Our assay revealed a sufficient amount of polyphenols in testa and pericarps of the tested wheat grains, which yielded positive staining with ammoniacal silver. This approach allowed us to detect deposition of the polyphenols in cell walls of the wheat grains pericarp with the bound phenolic acids among which dehydrodiferulates have been reported as predominant (Parker et al., 2005). Abundant colorless polyphenols were also detected by ammoniacal silver staining in seed envelopes of vetch; this feature may affect feeding properties of this crop and deserves deeper research too. Unlike purple pigments in the barley pericarp, the purple pigments in the wheat pericarp are insoluble and are not washed out by hot ethanol; however they alter their color after a pH change, thereby enabling us to identify these purple pigments as anthocyanins. Previously, cyanidin-based compounds have been identified in the wheat pericarp as major pigments, among which cyanidin-3-glucoside, cyanidin-3-rutinoside, and peonidin-3-glucoside are predominant (Abdel-Aal et al., 2006; Ficco et al., 2014). Insoluble blue pigments have also been observed in the aleurone layer of wheat and in macrosclereids of vetch. They have been identified as anthocyanins owing to their color transition from blue (original color) to red in an acidic medium. In blue-grained wheat, delphinidin-derived anthocyanins – delphinidin-3-glucoside and delphinidin-3-rutinoside – have been identified as predominant anthocyanins (Knievel et al., 2009; Abdel-Aal et al., 2016), whereas in vetch, the precise chemical structure of the blue pigments has not been determined yet. Judging by the results of our test, we can predict that vetch grains accumulate similar blue delphinidin-derived anthocyanins.

It also should be noted that differential behavior of anthocyanins during hot ethanol treatment may be attributed not only to their distinct chemical structure but also to the type of cells and organelles accumulating these pigments. For example, in wheat, blue anthocyanins accumulate in aleurone cells’ vacuoles. There are spherical particles called aleurone granules in aleurone cells, which are either phytate inclusions (type 1: composed of phytic acid minerals) or niacin inclusions (type 2: composed of niacin and proteins), each granule being surrounded by a fine layer of lipidic droplets (Morrison et al., 1975; Yu et al., 2021). The anthocyanins accumulated in aleurone cells may form complexes with metal ions or interact with the proteins; both these properties are attributed to anthocyanins and reportedly improve stability of the molecules (Li et al., 2021).

## Conclusion

In the current work, the histochemical assay consisting of a series of standard chemical tests applied to cryosections followed by microscopic examination successfully showed the possibility of distinguishing polyphenolic pigments and uncolored polyphenols in barley, wheat, and vetch grains. However, the extrapolation of results obtained on barley (and confirmed by analytical chemistry approaches) to other species requires caution, and further chemical analyses are necessary for exact identification of polyphenols. Nevertheless, the proposed histochemical assay has a high potential for polyphenolic profiling as part of preliminary screening of grains of barley and many other species where only a single grain is required. After the proposed preliminary screening, the selected lines/varieties could be propagated and analyzed in more detail for the presence of certain chemicals by more precise quantitative and qualitative methods.

## Conflict of interest

The authors declare no conflict of interest.
